# *In situ* neutron diffraction for analysing complex coarse-grained functional materials

**DOI:** 10.1107/S1600576723005940

**Published:** 2023-08-01

**Authors:** Manuel Hinterstein, Lucas Lemos da Silva, Michael Knapp, Alexander Schoekel, Martin Etter, Andrew Studer

**Affiliations:** a Fraunhofer IWM, Freiburg, Germany; b Institute for Applied Materials, Karlsruhe Institute of Technology, Karlsruhe, Germany; c Deutsches Elektronensynchrotron DESY, Hamburg, Germany; d Australian Nuclear Science and Technology Organisation, Sydney, Australia; Australian Synchrotron, ANSTO, Australia

**Keywords:** neutron diffraction, *in situ*, applied electric fields, barium titanate, strain mechanisms, grain sizes, complex functional materials, microstructures, coexisting phases

## Abstract

Complex functional materials play a crucial role in a broad range of energy-related applications and in general for materials science. Revealing the structural mechanisms is challenging due to highly correlated coexisting phases and microstructures, especially for *in situ* or *operando* investigations. Since the grain sizes influence the properties, these microstructural features further complicate investigations at synchrotrons due to the limitations of illuminated sample volumes. In this study, it is demonstrated that such complex functional materials with highly correlated coexisting phases can be investigated under *in situ* conditions with neutron diffraction. For large grain sizes, these experiments are valuable methods to reveal the structural mechanisms. For an example of *in situ* experiments on barium titanate with an applied electric field, details of the electric-field-induced phase transformation depending on grain size and frequency are revealed. The results uncover the strain mechanisms in barium titanate and elucidate the complex interplay of stresses in relation to grain sizes as well as domain-wall densities and mobilities.

## Abstract 2

This work reports *in situ* neutron diffraction experiments on a broad range of grain sizes of barium titanate. The study reveals the grain-size-dependent strain mechanisms and shows the competitiveness of neutron diffraction with high-resolution synchrotron diffraction.

## 1. Introduction

Complex functional materials may contain a whole range of real-structure effects, which influence the material properties and thus have an impact on their functionality, reliability and service life. These real-structure effects range from impurities or dopants through dislocations to segregations and space charge zones at the grain boundaries. In most cases, the microstructures of metallic functional materials consist of a broad range of such effects (Gottstein, 2007 ▸). In the case of single-phase materials (*e.g.* electrical steel, electrolytic copper, α-brass, pure iron), point defects (substitution atoms, interstitial atoms, diffusion), line defects (dislocations and their influence on deformation) and surface defects (twins, antiphase boundaries and stacking faults), in addition to grain boundaries, play a major role. In the case of technical alloys, highly correlated coexisting phases dominate the microstructures, such as in eutectic alloys (Al cast alloys) (Yan *et al.*, 2020 ▸), duplex- (Knyazeva & Pohl, 2013 ▸) or dual-phase steels (Szewczyk & Gurland, 1982 ▸), and the common Ti alloy TiAl_6_V_4_ (Galindo-Fernández *et al.*, 2018 ▸).

Such highly correlated phase coexistences might be found in functional ceramic materials as well. One of the most well known material systems is lead zirconate titanate (PZT) solid solution, where the most interesting compositions are located in the vicinity of a composition-dependent phase boundary (Noheda, 2002 ▸). In this compositional range, the desirable properties are enhanced due to these phase coexistences (Hinterstein *et al.*, 2015 ▸). Such coexistences of highly correlated phases are reported in a range of functional ceramic material systems, such as PMN–PT [*x*PbMg_1/3_Nb_2/3_O_3_–(1 − *x*)PbTiO_3_] (Noheda *et al.*, 2002 ▸), KNN (K_*x*_Na_1−*x*_NbO_3_) (Zhang *et al.*, 2022 ▸) and NBT–BT [*x*Na_0.5_Bi_0.5_TiO_3_–(1 − *x*)BaTiO_3_] (Paterson *et al.*, 2018 ▸). For these material systems, controversial debates in the literature are continuing about the crystal structures at the phase boundaries. In most investigated systems, it is still unclear whether monoclinic phases exist or not. Other explanations involve coherence effects during the measurements (Wang, 2007 ▸) or complex chemical distributions (Hinterstein *et al.*, 2018 ▸), which both might be misinterpreted as single-phase monoclinic structures. The phase composition and the properties in these ferroelectric materials are also dependent on the grain size, as recently documented for PZT (Picht *et al.*, 2020 ▸) and barium titanate (BaTiO_3_, BT) (Lemos da Silva & Hinterstein, 2022 ▸; Lemos da Silva *et al.*, 2021 ▸; Buscaglia & Randall, 2020 ▸). Recent findings indicate that phase coexistences can even play a role in classical end members of phase diagrams such as BT (Shin, 2021 ▸; Lemos da Silva *et al.*, 2021 ▸).

Since BT is considered an ideal material system due to its simple *AB*O_3_ perovskite structure without any substitution or doping, it represents the classical model ferroelectric system. However, recent research has indicated that the BT system exhibits complex structural mechanisms as well. A pronounced change in functional properties as a function of grain size can be observed in BT (Buscaglia & Randall, 2020 ▸; Lemos da Silva *et al.*, 2021 ▸) as well as other ferroelectric systems such as PZT (Picht *et al.*, 2020 ▸). One of the reasons for this is the increasing intergranular stresses, stresses at the domain walls and the domain-wall mobility. A direct proof of the influence of stresses on the phase-transformation temperature was outlined by Schader *et al.* (2013 ▸) with uniaxial stresses in BT. The shift of the tetragonal to ortho­rhombic phase transformation temperature was determined to around 0.1 K MPa^−1^ in polycrystalline BT. Wang *et al.* (2014 ▸) demonstrated that a ferroelectric phase could be induced by applying an electric field several kelvins above the Curie temperature. At room temperature, indications of a phase coexistence were outlined by Kalyani *et al.* (2015 ▸) with careful analysis of high-resolution X-ray and neutron diffraction data. Here, the first indications of an orthorhombic phase at room temperature appeared. These features became more apparent with *in situ* experiments with applied electric fields (Ghosh *et al.*, 2014 ▸). However, clear proof of a field-induced phase transformation could only be delivered by high-resolution *in situ* synchrotron experiments with a multi-analyser detector (MAD) (Schökel *et al.*, 2021 ▸) by Lemos da Silva *et al.* (2021 ▸). With a quantitative analysis, using the STRAP method (strain, texture and Rietveld analysis for piezoceramics) (Hinterstein *et al.*, 2019 ▸, 2015 ▸), the phase fractions and the individual strain mechanisms could be determined. The STRAP method is able to quantify strain contributions from the crystal structure itself, as well as from domain switching and lattice strain from the converse piezoelectric effect.

Knowledge about the structural details in functional materials is crucial for understanding the functional mechanisms, as well as for developing and tailoring next-generation sustainable materials with new or improved functionalities. In order to reveal the details about the structural mechanisms, sophisticated characterization methods are necessary. The most common technique is powder diffraction with either X-rays or neutrons. For coexistences of highly correlated phases, high angular resolution is crucial to resolve the subtle structural differences. At the same time, details about the structural mechanisms during operation can only be determined from *in situ* or *operando* experiments. A great example for this is the recently revealed electric-field-induced phase transformation in BT (Lemos da Silva *et al.*, 2021 ▸). The clear splitting of the reflections could only be observed with a MAD (Schökel *et al.*, 2021 ▸). For X-ray diffraction, *in situ* or *operando* experiments with high angular resolution usually involve synchrotron radiation of high energy (>40 keV) to overcome limitations which arise from absorption (Ehrenberg *et al.*, 2013 ▸, 2019 ▸). The combination of high angular resolution and high energy is rarely optimized, but can be found at specialized beamlines such as 11BM at the Advanced Photon Source (Wang *et al.*, 2008 ▸), MSPD at ALBA (Fauth *et al.*, 2013 ▸; Peral *et al.*, 2011 ▸), P02.1 at PETRA III (Herklotz *et al.*, 2013 ▸; Dippel *et al.*, 2015 ▸) or ID22 at ESRF (Dejoie *et al.*, 2018 ▸; Fitch, 2004 ▸).

However, due to the ever-increasing brilliance in next-generation synchrotrons, new challenges arise. Modern third-generation and future fourth-generation synchrotrons exhibit high brilliance from small source sizes (Shin, 2021 ▸). These properties are perfect for building beamlines with high angular resolution or for focusing down to the nanometre scale. On the other hand, practical limitations for the samples arise. Due to the significantly reduced divergence, the diffraction condition must be fulfilled precisely to result in significant reflection intensities. Therefore, not all crystallites in a sample might contribute to the diffraction pattern. With sample spinning in capillary geometries, this can be avoided. However, for *in situ* or *operando* experiments this strategy is often not feasible. Therefore, grain statistics might be the most important limitation of experiments. With small beam sizes and limited sample thicknesses, samples with microstructures consisting of large grains might not be suitable for *in situ* or *operando* experiments anymore. Since some functional properties depend on grain sizes, this is a major limitation for materials science beamlines.

Neutron powder diffraction offers the possibility of low absorption effects, which allows complex sample environments and large samples for *in situ* or *operando* experiments. Therefore, grain sizes impose no limitation in most cases. Time-of-flight beamlines such as HRPD at ISIS (Ibberson, 2009 ▸) provide very high angular resolutions. Constant-wavelength beamlines can usually not compete with such high-resolution beamlines. However, due to the large diffraction angles in the monochromator and the property of neutron diffraction that reflection intensities are preserved for high-indexed reflections, the information from neutron experiments has other advantages. Neutron beamlines like SPODI at the Heinz Maier-Leibnitz Zentrum (MLZ) (Hoelzel *et al.*, 2012 ▸), D2B or D20 at the Institut Laue–Langevin (ILL) (Hansen *et al.*, 2008 ▸), or Wombat at the Australian Nuclear Science and Technology Organisation (ANSTO) (Studer *et al.*, 2006 ▸) provide a combination of versatility and high angular resolution. Beamlines D20 and Wombat additionally exhibit the capability to perform stroboscopic experiments with high time resolutions down to the microsecond regime (Hinterstein *et al.*, 2023 ▸).

For future materials science studies under external stimuli or in other forms of *in situ* or *operando* experiments on materials with grain sizes well above several micrometres, neutron diffraction might be the only choice to get diffraction data with good statistics for quantitative analysis. In order to obtain experimental proof that fast materials science neutron beamlines are able to resolve complex crystal structures and deliver high-quality diffraction data for quantitative analysis, we compared different beamlines and performed *in situ* experiments on the challenging material BT in this study.

## 2. Experimental

Instrumental resolution functions were determined from several materials science beamlines at synchrotron and neutron sources. In order to determine the instrumental resolution function, profile standard samples were measured. This was either LaB_6_ or Na_2_Al_2_Ca_3_F_14_. Rietveld refinement of the diffraction data was performed using the *Fullprof* software package (Rodríguez-Carvajal, 1993 ▸). In order to obtain the instrumental resolution function, profile parameters were refined together with wavelength, background and scale parameters.

At the synchrotron sources, three different detector types were used. For the highest angular resolution, 0D detectors with analyser crystals were used. Intermediate-angular-resolution data were collected with 1D strip detectors and low-angular-resolution data were acquired from 2D panel detectors. The 1D data were collected with Mythen strip detectors (Schmitt *et al.*, 2003 ▸) with a strip size of 50 µm. At the MS beamline of the Swiss Light Source (SLS) (Patterson *et al.*, 2005 ▸), a wavelength of 0.44288 Å was used with a sample-to-detector distance of 760 mm. At the MSPD beamline at ALBA (Fauth *et al.*, 2013 ▸), a wavelength of 0.41323 Å was used with a sample-to-detector distance of 550 mm. Zero-dimensional and two-dimensional data were collected at the P02.1 beamline at PETRA III (Dippel *et al.*, 2015 ▸; Herklotz *et al.*, 2013 ▸) at a wavelength of 0.20703 Å. The high-angular-resolution data were collected with a Si 111 MAD (Schökel *et al.*, 2021 ▸), while the 2D data were collected with a Perkin­Elmer 1621N ES detector at distances of 1200 and 2200 mm (Herklotz *et al.*, 2013 ▸).

At the neutron sources, data were collected with banana-shaped multi-detector arrays that cover a broad angular range. The detection principle is based on ^3^He, which results in a 2D position-sensitive detector that is effectively used as a 1D detector in most cases. The SPODI beamline at MLZ (Hoelzel *et al.*, 2012 ▸) was operated at 1.54828 Å. Here, its 80 detector modules cover a 2θ range of 160° and are positioned in 40 resolution steps to get an effective pixel size of 0.05° in 2θ. The D20 beamline at ILL (Hansen *et al.*, 2008 ▸) was operated at 1.54334 and 2.41703 Å. Its detector array covers a 2θ range of 153.6° with 1536 strips, resulting in an effective strip width of 0.1° in 2θ. The Wombat beamline at ANSTO (Studer *et al.*, 2006 ▸) was operated at 1.49739, 1.63742 and 2.41855 Å. Its position-sensitive detector covers a 2θ of 120° with an effective pixel size of 0.1° in 2θ.

Besides this, the instrumental resolution for selected neutron and synchrotron-based diffraction instruments was taken from the *FullProf* repository of instrument resolution files (Rodríguez-Carvajal, 1993 ▸), *e.g.* ID31 (ESRF) equipped with a MAD (Dejoie *et al.*, 2018 ▸; Fitch, 2004 ▸) at a wavelength of 0.336367 Å, the D2B diffractometer at ILL in high-resolution configuration (α_1_ = 5′) at a wavelength of 1.594216 Å (Suard & Hewat, 2001 ▸) and the HRPD instrument at ISIS using the high-resolution backscattering detector bank (Ibberson, 2009 ▸).

BT was prepared by the solid-state route as described elsewhere (Lemos da Silva *et al.*, 2021 ▸) from ceramic powder (Alfa Aesar, 99%). Samples were uniaxially pressed at 30 MPa and compacted with a cold isostatic press at 400 MPa. Different grain sizes were adjusted with different sintering techniques as described elsewhere (Lemos da Silva *et al.*, 2021 ▸). For *in situ* neutron diffraction experiments at the Wombat beamline at ANSTO, samples were cut into rectangular bars of 3.5 × 3.5 × 20 mm. Electrodes were painted with silver paste and samples were contacted in a special sample environment for applying electric fields (Simons *et al.*, 2014 ▸). Electric fields were applied up to 2 kV mm^−1^ and diffraction patterns were collected at different ω positions from 0 to 180°. Quantitative data analysis was performed with the program package *MAUD* (Grässlin *et al.*, 2013 ▸). Data analysis was performed with the STRAP method as described elsewhere (Hinterstein *et al.*, 2019 ▸, 2015 ▸). With this method an orientation series is used for the refinement of a structure model together with a texture and strain model, in order to quantitatively determine the strain mechanisms.

## 3. Results and discussion

**Figure 1:**
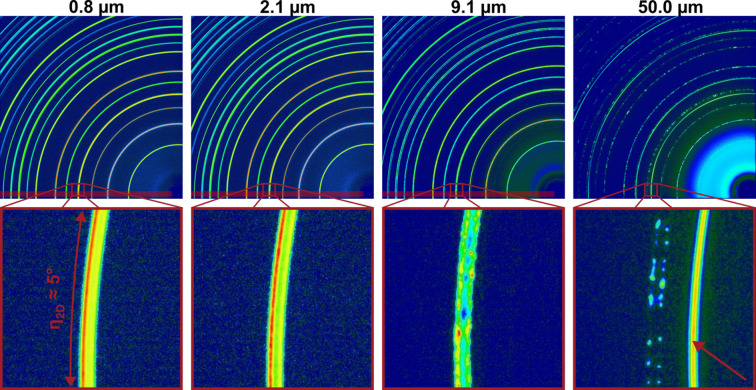
Two-dimensional diffraction patterns from the P02.1 beamline at PETRA III at a high-angular-resolution sample-to-detector distance of 2200 mm for samples with 0.8, 2.1, 9.1 and 50.0 µm grain size (top row). Magnification of the 200 reflection for the different grain sizes (bottom row). The sample with a grain size of 50.0 µm additionally exhibits electrode reflections, marked with a red arrow. The transparent red region indicates the opening window of the MAD during a 2θ scan.

In an earlier study, we were able to uncover a field-induced phase transformation in BT for grain sizes of 0.8 and 2.1 µm (Lemos da Silva *et al.*, 2021 ▸). Fig. 1 ▸ shows the 2D diffraction patterns of these two samples together with two samples with grain sizes of 9.1 and 50.0 µm. The data were collected at the high-angular-resolution sample-to-detector distance of 2200 mm at beamline P02.1 at PETRA III. With this setup it was not possible to quantify the field-induced phase transformation with the STRAP method from the 2D data. The 2D data illustrate the effect of coarse grains on the diffraction patterns. A more detailed view of the characteristic 200 reflection is provided in the magnified bottom-row images. The 200 reflection shows continuous diffraction rings with smooth intensity distributions, especially for the sample with a grain size of 0.8 µm. The sample with a grain size of 2.1 µm shows a distinct granularity in the intensity distribution, especially for the 200_T_ reflection. (The subscript T denotes tetragonal indexing.)

The two examples with coarse grain sizes have a different appearance. The sample with 9.1 µm grain size exhibits a spotty intensity distribution along the diffraction rings. For the sample with a grain size of 50.0 µm, just a few grains contribute to the diffraction rings, resulting in isolated intensities along the diffraction ring. The continuous diffraction ring marked with a red arrow originates from a silver electrode made from silver paste with fine particles. The magnifications in Fig. 1 ▸ depict a detector slice at the 200 reflections of η_2D_ ≃ 5°, where η_2D_ is the azimuthal angle on the 2D detector around the primary beam. The typical detector opening (acceptance angle perpendicular to the diffraction plane) of a MAD detector is between 1 and 3° (Schökel *et al.*, 2021 ▸). This acceptance angle can be compared with η_2D_ and is indicated as a transparent bar in Fig. 1 ▸. This illustrates that the sample with 9.1 µm grain size is no longer suitable for high-angular-resolution measurements at this beamline with a MAD detector, since it cannot be guaranteed that the resulting diffraction pattern measured with the detector window represents the correct reflection intensity ratios. Therefore, grain-size-dependent studies cannot be performed towards coarse grain sizes. These issues with grain statistics could be compensated with a 2D detector, where the opening angle can be varied by increasing the range of integration over η_2D_. However, since the angular resolution of the 2D detector was not sufficient for these experiments, an *in situ* investigation of BT was not feasible for coarse-grained samples with synchrotron radiation.

**Figure 2:**
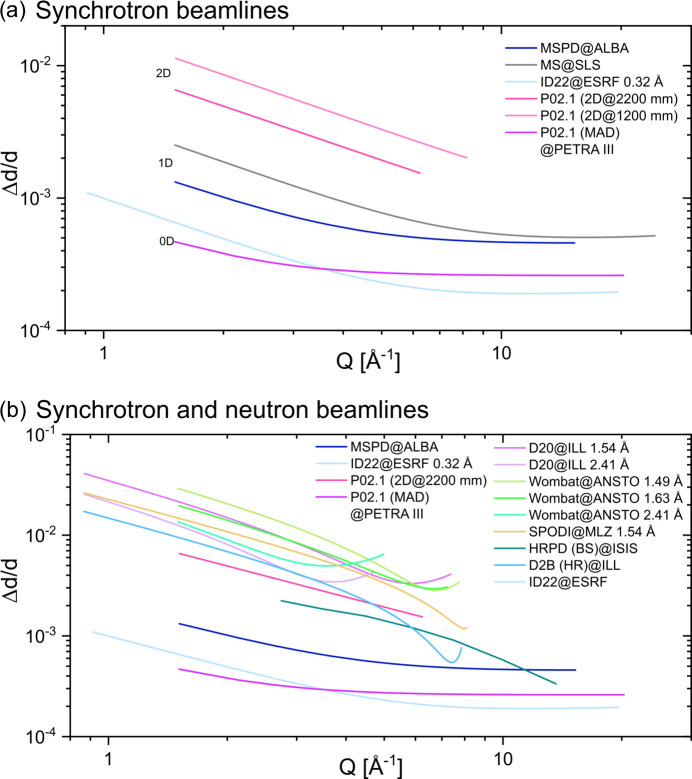
(*a*) Instrumental resolution functions, measured with either LaB_6_ or Na_2_Al_2_Ca_3_F_14_, for synchrotron beamlines with different detectors (0D, 1D, 2D). (*b*) Comparison of the instrumental resolution functions for selected detectors of synchrotron beamlines with high-resolution neutron beamlines for materials science experiments.

Fig. 2 ▸(*a*) illustrates the instrumental angular resolution functions for different detector types at synchrotron beamlines. Since the graph compares beamlines at different wavelengths ranging from 30 to 60 keV photon energies, the resolution functions are plotted as a function of the scattering vector magnitude *Q*. In order to be able to compare the significantly differing values, a double logarithmic scale is used. As expected, the 0D MAD detectors at beamlines P02.1 at PETRA III and ID22 at ESRF show the highest angular resolution, close to the physical limit. The 1D Mythen detectors at the MS beamline of the SLS and the MSPD beamline of ALBA show good intermediate-angular-resolution functions. Since these detectors have 1280 channels per module (strips) and usually several modules, or even much more (24 modules with 30 720 strips in the case of the MS beamline at SLS), the acquisition times are orders of magnitude shorter than those of 0D detectors. However, due to the sensor material (typically silicon) and thickness (typically several hundred micrometres), the detectors are practically limited to maximum photon energies of around 30 keV, which is not sufficient for *in situ* experiments in transmission geometry. Two-dimensional detectors such as those used in Fig. 1 ▸ have larger pixel sizes and thus can be operated at high photon energies, and have short acquisition times due to the large number of pixels (>4 MP), with the cost of significantly reduced angular resolution.

Fig. 2 ▸(*b*) compares the instrumental angular resolution functions of the 0D, 1D and 2D detectors with those of neutron diffraction beamlines. The angular-resolution function of the HRPD time-of-flight beamline in backscattering setup (BS) at ISIS is superior to those of the rest of the neutron beamlines, and even reaches the same range as the 0D detector of the P02.1 beamline at PETRA III and the ID31 beamline at ESRF. However, most *in situ* experiments are performed at constant-wavelength beamlines, where the necessary infrastructure for such studies already exists. The angular-resolution functions of the SPODI beamline at MLZ, the D20 beamline at ILL and the Wombat beamline at ANSTO are all in a similar range and are comparable to the resolution functions of the 2D detector at the P02.1 beamline at PETRA III. This indicates that, similar to the 2D results at P02.1, *in situ* experiments with BT samples are not feasible due to a lack of angular resolution (Lemos da Silva *et al.*, 2021 ▸). However, the intensities in neutron diffraction experiments exhibit a significantly different distribution from those at X-ray experiments. In neutron experiments, the intensities of high-indexed reflections are significantly higher. This is because the scattering length *b* in neutron scattering is essentially independent of the scattering angle. Additionally, the resolution functions exhibit a pronounced minimum at high diffraction angles, especially for carefully selected monochromator angles, as in the case for the 1.49 Å setup of the Wombat beamline or the 1.54 Å setup of the SPODI beamline [Fig. 2 ▸(*b*)]. This minimum of the resolution function depends on the diffraction angles of the monochromator. It was already shown in a previous study that the data from SPODI can reveal similar structural details to high-resolution synchrotron data with a 0D analyser detector, when analysing the high-indexed reflections (Hinterstein *et al.*, 2018 ▸).

In order to find out if *in situ* neutron diffraction can also reveal the structural responses in complex functional materials, we performed *in situ* experiments with an applied electric field on BT at the Wombat beamline at ANSTO, similar to what we previously reported with synchrotron experiments (Lemos da Silva *et al.*, 2021 ▸). However, due to the larger sample volume, we were able to investigate a broad range of grain sizes, namely 0.8, 2.1 and 14.8 µm. As can be seen from Fig. 1 ▸, a synchrotron experiment for the sample with a grain size of 14.8 µm would clearly not be feasible.

**Figure 3:**
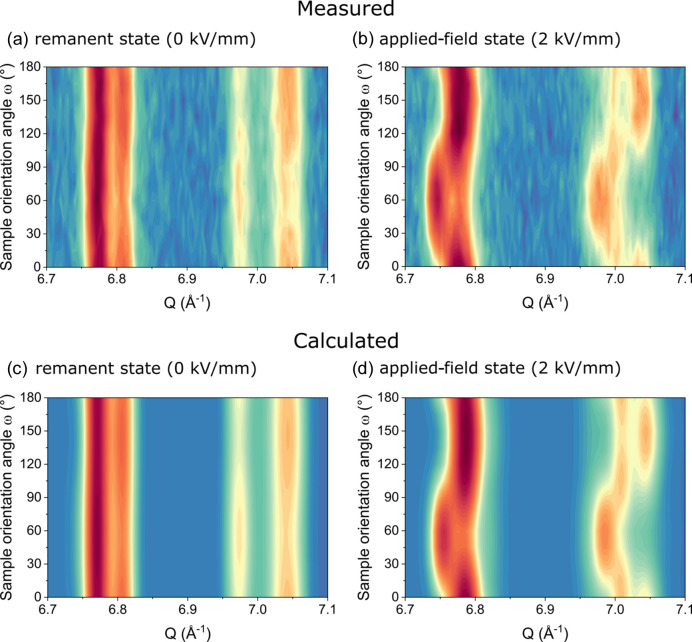
Sample orientation series from neutron diffraction at Wombat with a wavelength of 1.49 Å for the sample with a grain size of 14.8 µm. Measured diffraction patterns of the 420 and 421 reflections in (*a*) the remanent state at 0 kV mm^−1^ and (*b*) the applied-field state at 2 kV mm^−1^. Calculated diffraction patterns of the 420 and 421 reflections in (*c*) the remanent state at 0 kV mm^−1^ and (*d*) the applied-field state at 2 kV mm^−1^.

Figs. 3 ▸ and 4 ▸ show measured diffraction patterns and corresponding refinements with a two-phase structure model of a tetragonal *P*4*mm* phase and an orthorhombic *Amm*2 phase of the sample with a grain size of 14.8 µm. Due to the significantly larger sample of 3.5 × 3.5 × 20 mm, which is completely submerged in the neutron beam, grain statistics play no role, even for these relatively large grain sizes. The selected range depicts the 420 and 421 reflections, which lie in the range of the best angular resolution of the Wombat beamline in the 1.49 Å setup. Figs. 3 ▸(*b*), 3 ▸(*d*) and 4 ▸(*b*) show that the angular resolution is high enough to resolve the pronounced and complex structural response to the applied electric field. Figs. 3 ▸(*c*) and 3 ▸(*d*), as well as 4 ▸(*a*) and 4 ▸(*b*), demonstrate that the quantitative analysis with the STRAP method yields a highly accurate fit, which is able to reproduce all structural features of the measurements.

**Figure 4:**
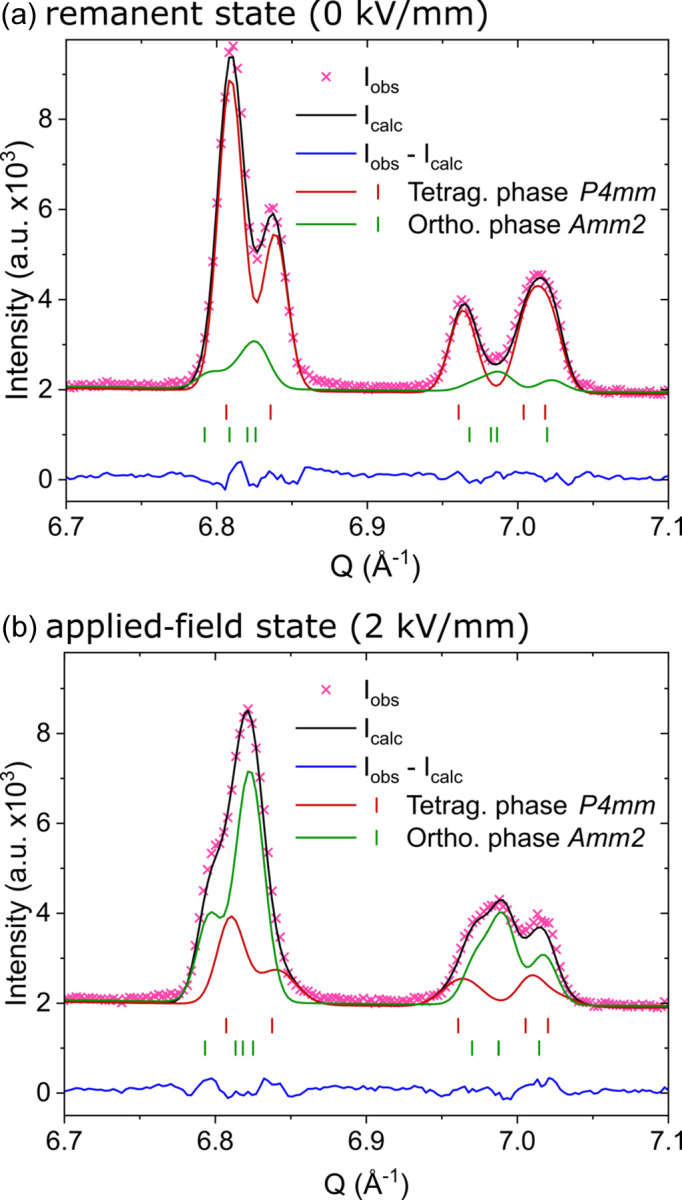
Selected range of the Rietveld refinement with neutron diffraction data from Wombat with a wavelength of 1.49 Å of the 420 and 421 reflections in (*a*) the remanent state at 0 kV mm^−1^ and (*b*) the applied-field state at 2 kV mm^−1^. A refinement with a two-phase structure model of a tetragonal *P*4*mm* phase and an orthorhombic *Amm*2 phase was carried out. The refinement shows a superposition of all measured sample orientations.

The superposition of all measured orientations in Fig. 4 ▸ might indicate that the angular resolution is not high enough to accurately distinguish between the tetragonal and the orthorhombic phase. However, the details in Figs. 3 ▸(*b*) and 3 ▸(*d*) show that the individual phases appear with different intensities at different sample orientation angles. This is again a confirmation that phase coexistences play a crucial role for the electromechanical response in piezoceramics. The differently oriented grains in the polycrystalline material respond in different ways, depending on their orientation with respect to the applied electric field direction. This way the material is able to increase the response to an applied electric field, since more directions for the polarization direction are accessible. We recently confirmed this with phase field simulations on PZT (Fan *et al.*, 2022 ▸). Since the refinements are excellent considering the quality of the measured data, *in situ* measurements at the Wombat beamline are an alternative to *in situ* synchrotron measurements when the grain sizes exceed the feasibility limit.

**Figure 5:**
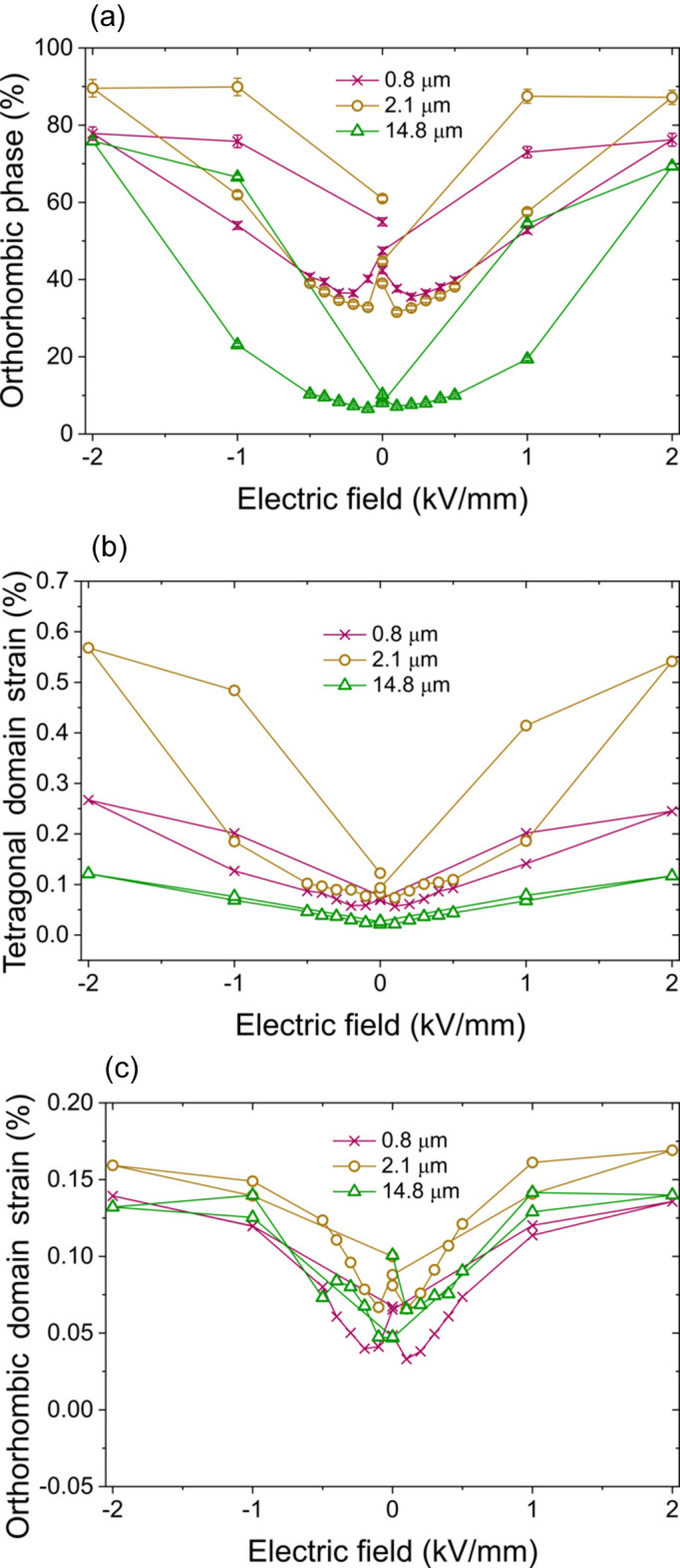
Refinement results of the analysis with the STRAP method with a two-phase structure model of a tetragonal *P*4*mm* phase and an orthorhombic *Amm*2 phase. (*a*) Orthorhombic phase fraction, and (*b*) tetragonal and (*c*) orthorhombic domain switching strain for samples with grain sizes of 0.8, 2.1 and 14.8 µm.

Figs. 3 ▸ and 4 ▸ indicate a pronounced response of the sample with 14.8 µm grain size. This is confirmed by the quantitative analysis of the data with the STRAP method. The results are shown in Fig. 5 ▸. The phase fractions in Fig. 5 ▸(*a*) illustrate that the field-induced phase transformation of the coarse-grained 14.8 µm sample is the largest. In the remanent state at 0 kV mm^−1^, the sample appears almost purely tetragonal with an orthorhombic phase fraction below 10%. With applied field, the phase fraction increases continuously and reaches almost 80% at 2 kV mm^−1^. With a change of phase fraction of almost 70% this is, to our knowledge, the largest amplitude of reversible field-induced phase transformation. The sample with a grain size of 2.1 µm still reaches an amplitude of around 50%. The sample with the smallest grain size of 0.8 µm has a total amplitude of around 40% and shows a distinct minimum at the coercive field. This indicates a fundamental change in the electric-field-dependent strain behaviour with decreasing grain size.

Figs. 5 ▸(*b*) and 5 ▸(*c*) depict the domain switching strains calculated from the STRAP analysis for the two individual phases. When comparing the strain mechanisms of the two phases, significant differences appear. The orthorhombic strain hystereses of all three samples appear similar with slightly different levels of remanent strain [Fig. 5 ▸(*c*)]. While the strain loop of the sample with a grain size of 14.8 µm shows almost no hysteresis at all, the strain loop of the sample with 0.8 µm grain size shows distinct negative strain around the coercive field and a significant hysteresis.

The tetragonal strain loops in Fig. 5 ▸(*b*) show a completely different appearance. The overall level of remanence is significantly lower. While the sample with a grain size of 14.8 µm shows almost no remanent strain, the other two samples show a significantly higher remanent strain. The scales of Figs. 5 ▸(*b*) and 5 ▸(*c*) deviate from each other significantly. This is especially due to the enormous tetragonal domain switching strain of the sample with a grain size of 2.1 µm. The reason here is the extremely low tetragonal phase fraction of under 10% [see Fig. 5 ▸(*a*)]. This results in low reflection intensities of the tetragonal phase, and thus significant uncertainties in calculating the domain texturing and with that the domain switching strain. The tetragonal domain switching strain loops show the same behaviour as for the orthorhombic phase with a significantly increasing hysteresis towards smaller grain sizes and distinct strain behaviour around the coercive field.

**Figure 6:**
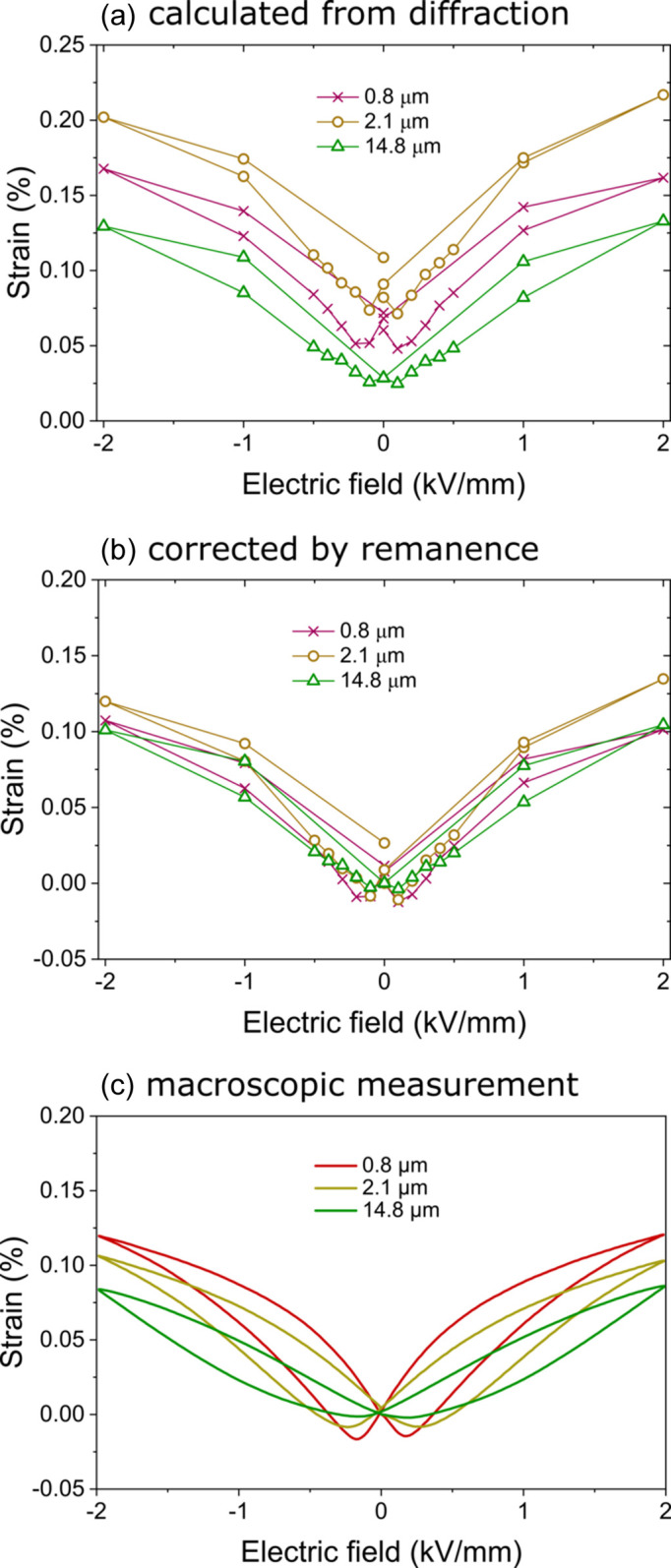
Strain hystereses for the samples with grain sizes of 0.8, 2.1 and 14.8 µm. (*a*) Calculated from diffraction with the STRAP method, and (*b*) calculated from diffraction with the STRAP method and corrected by the remanent values to compare the strain hystereses with (*c*) the macroscopically measured strain hystereses.

The STRAP method allows one to calculate the resulting macroscopic strain hysteresis from the phase fractions and the individual strain mechanisms. Our detailed previous work revealed that BT exhibits strong domain switching strain but no lattice strain (Lemos da Silva *et al.*, 2021 ▸). Since this previous study is based on MAD data with the highest possible angular resolution, the reflection shifts that are characteristic for lattice strain can be evaluated with high precision. However, no apparent reflection shifts as a function of orientation angle could be observed. Therefore, the main strain mechanisms are the domain switching strains of the individual phases. Fig. 6 ▸(*a*) shows the resulting calculated strain hystereses for the different grain sizes. Fig. 6 ▸(*b*) shows the same strain hystereses corrected by the remanent values at 0 kV mm^−1^ for better comparison with the macroscopic strain hystereses in Fig. 6 ▸(*c*). While the macroscopic measurements show distinct differences in strain amplitude and shape of the strain hystereses, the strain loops calculated from diffraction appear almost identical. This is especially surprising when considering the strong differences in the phase fractions [Fig. 5 ▸(*a*)] and the tetragonal domain switching strain [Fig. 5 ▸(*b*)]. We have already reported such complex strain mechanisms adding up to rather simple strain loops in a lead-free NBT–BT composition (Lee *et al.*, 2020*a* ▸,*b* ▸). As reported there, the frequency plays an important role for the appearance of the strain loops. While the macroscopic measurements in Fig. 6 ▸(*c*) were performed at 10 Hz, the neutron diffraction experiment took almost a whole day, which resulted in an effective frequency of around 10 µHz. This results in a difference of around six orders of magnitude in frequency, which explains the significant differences in appearance of the strain loops.

When comparing the strain hystereses in Figs. 6 ▸(*a*) and 6 ▸(*b*) of the samples with different grain sizes, the pronounced negative strain at the coercive field of the sample with a grain size of 0.8 µm becomes apparent. For the sample with a grain size of 2.1 µm this feature is only visible for one measurement point, and the sample with a grain size of 14.8 µm shows no negative strain at all. This is in good agreement with the macroscopic measurements [Fig. 6 ▸(*c*)], despite the greatly differing frequencies of the two experiments. Fig. 6 ▸(*b*) shows the three strain loops corrected by the remanent strain at 0 kV mm^−1^. This comparison illustrates that the strain amplitude of all three samples is very similar. Only the sample with a grain size of 2.1 µm exhibits a slightly higher strain. When comparing the strain loops in Fig. 6 ▸(*c*) from the macroscopic measurements, the three samples show distinct differences. Here, the strain amplitude increases with decreasing grain size.

The differences between the diffraction experiments and the macroscopic measurements can be explained by the strongly different measurement frequencies. The 10 Hz of the macroscopic measurements does not allow slow processes to contribute to the strain loops. As we have already shown for PZT (Hinterstein *et al.*, 2019 ▸) and NBT–BT (Lee *et al.*, 2020*a* ▸,*b* ▸), the strain mechanisms change significantly when varying the frequency towards the millihertz or microhertz regime. This process has been reported as ferroelectric creep (Zhou & Kamlah, 2006 ▸). However, this creep has always been reported for solid solutions, where composition-dependent phases coexist. With BT, this is the first time that such a process has been identified in a single-component material. The grain-size dependence has also never been investigated.

The strain amplitudes of the sample with a grain size of 0.8 µm are almost identical in both the macroscopic and the diffraction experiment. This indicates that the time-dependent response in this fine-grained sample does not vary strongly. The strain mechanisms have a quick response with full amplitude in the sub-second range. This might be due to the high domain-wall density and high domain-wall mobility. This is the explanation of the maximum in properties for grain sizes in the range of 1 µm. A frequency dependence over a broad range of frequencies towards millihertz and microhertz was never investigated due to the fact that such measurements become extremely challenging macroscopically because of external influences such as vibrations, drift or temperature variations. Therefore, the results here are extremely valuable to understand the grain-size-dependent properties. From Fig. 6 ▸(*b*) it is obvious that for very slow frequencies, in the range of microhertz, the strain amplitudes become comparable. The shape of the strain loops is still the same as for macroscopic measurements at frequencies in the range of hertz, with the negative strain at the coercive field for small grain sizes. However, the amplitudes for large grain sizes increase significantly.

This indicates that, in large grains, the lower domain-wall density and mobility result in a significant deceleration of the strain mechanisms. Such effects of slow responses towards low frequencies have already been reported for PZT (Zhukov *et al.*, 2014 ▸) and BT-based compositions (Zhukov *et al.*, 2015 ▸). These experiments indicate that, whenever an electric field of sufficient intensity is applied, the maximum polarization will be reached. With decreasing electric field strength, the time to reach the maximum polarization may occur in the range of minutes or even hours. The results from Fig. 5 ▸(*a*) also indicate that for large grain sizes the field-induced phase transformation plays a more important role. In such a microstructure, the grain-boundary density decreases significantly and thus the stresses associated with a change of crystal structure can be accommodated more easily. It is already known that the orthorhombic tetragonal phase transformation temperature increases with decreasing grain size (Buscaglia & Randall, 2020 ▸). This explains the low orthorhombic phase fraction in the remanent state for the sample with a grain size of 14.8 µm [Fig. 5 ▸(*a*)]. One reason might be the increased stresses in the small grains, which we have already reported for PZT (Picht *et al.*, 2020 ▸) and BT (Lemos da Silva *et al.*, 2021 ▸). In BT, stresses can change the phase-transformation temperature significantly (Schader *et al.*, 2017 ▸). Together these effects explain the large phase-transformation amplitude for large grain sizes. Since these different strain mechanisms have different response times (Hinterstein *et al.*, 2023 ▸), for extremely slow frequencies the strain amplitudes are comparable.

With these experiments, the complementarity of high-resolution neutron beamlines with high-resolution synchrotron beamlines could be underlined. In addition, the large sample and beam sizes pose no limitations in terms of grain sizes in the investigated grain-size range. However, the classic neutron diffraction experiments are limited by the available frequencies. While synchrotron experiments with a 2D detector allow collecting all sample orientations in a single exposure and photon fluxes reduce exposure times to the second or sub-second range, quasistatic experiments can be performed in the range from microhertz to almost hertz. For neutron experiments, an orientation series has to be collected for each field step and a single data acquisition usually takes around 10 s to minutes. For the measurement of a full hysteresis, these experiments are limited to the microhertz range. However, neutron experiments can be performed stroboscopically, which allows one to access the hertz range as long as the material can be cycled reversibly for at least 10^5^ cycles (Hinterstein *et al.*, 2023 ▸). Due to technical limitations, the millihertz range is practically not accessible for neutron experiments.

This study clearly exposes the weaknesses and limitations of both synchrotron and neutron experiments. However, neutron experiments can clearly compete in terms of angular resolution with synchrotron experiments and even have significant advantages due to the large sample and beam sizes. The characterization of advanced functional materials with complex structure can be performed with unprecedented detail on a broad range of grain sizes. The results help in understanding the strain mechanisms and grain-size dependence in BT, which serves as an archetype ferroelectric material.

## 4. Conclusions

The results demonstrate that *in situ* neutron diffraction experiments are able to resolve even highly challenging structural mechanisms, which usually require the highest angular resolution for synchrotron experiments. Due to the large neutron beams and samples, this allows the investigation of coarse-grained functional materials with complex structure and microstructure. On the example of BT we were able to reveal the strain mechanisms over a broad range of grain sizes. The recently discovered field-induced phase transformation is highly grain-size dependent and is also dependent on frequency. The individual phases show distinctly different behaviour in their strain mechanisms. The interplay between the coexisting phases and their strain mechanisms together with the grain-size and frequency dependence uncovers the complex details of the electric-field-induced strain behaviour of BT.
